# Evaluation of the avian acute oral and sub-acute dietary toxicity test for pesticide registration

**DOI:** 10.1016/j.yrtph.2019.03.013

**Published:** 2019-03-25

**Authors:** Gina M. Hilton, Edward Odenkirchen, Melissa Panger, Garland Waleko, Anna Lowit, Amy J. Clippinger

**Affiliations:** a PETA International Science Consortium Ltd, London, UK; b United States Environmental Protection Agency, Washington, DC, USA^1^

## Abstract

The United States Environmental Protection Agency (USEPA), as well as other international regulatory agencies, require pesticide registrants to submit toxicity data that are used to conduct ecological risk assessments. While the USEPA has required both an acute oral and sub-acute dietary test in birds, trends in the use of data from these tests over the past 20 years have suggested that the avian sub-acute dietary test generally does not contribute to risk assessment conclusions. To address this question, a retrospective analysis was conducted to evaluate 119 pesticides with publicly available ecological risk assessments that were registered into commerce between 1998 and 2017. New pesticides (i.e., registered in the United States within the past 20 years) were chosen for the retrospective analysis to show utility of these tests for modern pesticide chemistries. Risk quotient (RQ) values (a point estimate of exposure divided by a deterministic toxicity endpoint) from the avian acute oral and dietary tests, as well as risk assessment conclusions, were compared to determine which test(s) drove the risk assessment findings. The RQ values were chosen as the data point for comparison in order to assess total risk (i.e., exposure and toxicity). After comparing RQ values from avian acute oral versus sub-acute dietary tests, there was only one case in which an avian sub-acute dietary RQ was greater than the acute oral RQ. Thus, the sub-acute dietary test did not identify risk in greater than 99% (118 out of 119) of chemicals based on results that either the acute oral RQ was higher than the sub-acute dietary RQ, or both the acute oral and the subacute dietary tests did not generate an RQ value of concern. For the one exception, both the oral and sub-acute RQ values were greater than the USEPA’s level of concern for endangered species. Based on the results of the retrospective analysis, it is concluded that in most cases avian risk can confidently be assessed without conducting the sub-acute dietary test.

## 1. Introduction

Data requirements for pesticides under title 40 Code of Federal Regulations (CRF) 158.630 for Ecological Effects require studies be submitted to the Office of Chemical Safety and Pollution Prevention (OCSPP) (EPA, 2017a) to fulfill the terrestrial and aquatic non-target organisms data requirements; including OCSPP series 850: Ecological Effects Test Guidelines (EPA, 2018a). The United States Environmental Protection Agency’s (USEPA) Environmental Fate and Effects Division (EFED), within the Office of Pesticide Programs (OPP), is responsible for assessing the environmental and ecological effects of conventional pesticides registered in the United States. For most pesticides used outdoors, the USEPA currently requires avian acute oral and subdietary toxicity tests for pesticide risk assessment following test guidelines 850.2100 (EPA, 2012a) and 850.2200 (EPA, 2012b), respectively. The Organisation for Economic Co-operation and Development (OECD) also has test guidelines for avian acute oral toxicity (OECD, 2010) and avian dietary toxicity testing (OECD, 1984).

The purpose of these tests is to determine a median lethal dose (LD_50_) or concentration (LC_50_) for the acute oral and sub-acute dietary toxicity tests, respectively. The sub-acute dietary test requires studies be conducted in upland game birds (e.g., northern bobwhite (*Colinus virginianus*)) and waterfowl (e.g., mallard duck (*Anas platyrhynchos*)). However, the acute oral test requires testing on both a passerine species (i.e., song bird), and either an upland game bird or a waterfowl. The acute oral toxicity test is conducted by a single administration of a test substance by capsule or gavage to a minimum of 10 birds per dosage level per species, followed by at least a 14-day observation period to monitor for mortality or signs of intoxication. In the sub-acute dietary test, the test substance is administered in the diet to 10 birds per dosage level for 5 days for each species, followed by at least 3 additional days of observation. The test guidelines describe the experimental design for both a definitive test and a limit test. The definitive test is designed to generate a median lethal dose (LD_50_) or concentration (LC_50_) response curve. A limit test is used for chemicals predicted to have relatively low toxicity. The limit test for the avian acute oral requires birds be dosed at ≥2000 mg active ingredient (a.i.)/kg-body weight (bw), and ≥5000 mg a.i./kg-diet dose is administered for the avian sub-acute dietary test (or the maximum expected environmental concentration, whichever is higher).

These data are used in the USEPA’s risk assessment process to help evaluate potential risks related to acute and sub-acute exposures in birds. Risk conclusions are generally formed around the evaluation of risk quotient (RQ) values in comparison to the level of concern (LOC). The LOC is used by the USEPA as a policy tool to indicate when a pesticide has the potential to cause adverse effects on non-target organisms as indicated when an RQ value is greater than one of the thresholds of interest: 0.5 for acute risk, 0.2 for acute restricted used, 0.1 for acute endangered species, and 1.0 for chronic risk (EPA, 2017b). The risk estimate comparison of RQ values to the LOC for the avian acute oral and sub-acute dietary test was used as the primary metric for assessing risk to avian terrestrial species. Specifically, acute avian RQ values are calculated by exposure, estimated environmental concentration (EEC), divided by a definitive toxicity value (RQ = EEC/LD_50_ or LC_50_, acute oral and sub-acute dietary, respectively); if an RQ value is greater than the LOC, then a risk concern is identified.

Preliminary evaluation of the avian risk conclusions suggested that the avian sub-acute dietary tests have not figured prominently into the USEPA’s risk assessments for new use chemicals registered between 1998 and 2017. Therefore, the aim of the retrospective analysis described herein was to generate a comparative study of the avian acute oral and sub-acute dietary RQ values in order to determine if the results of both tests are used by the USEPA to support risk assessment conclusions. Ultimately, analysis was conducted to examine the proportion of cases where the avian sub-acute dietary test is not considered a driver for risk assessments. The results of this retrospective analysis can be used by regulatory agencies to develop criteria for waiving the avian acute dietary test if appropriate, which could significantly reduce the number of animals used in the pesticide regulatory process while adequately protecting wildlife.

## 2. Methods

### 2.1. Chemical selection

For this retrospective analysis, the focus was on new conventional technical grade pesticides submitted to the USEPA for registration within the last 20 years. Newer chemistries were analyzed because those were assumed to be most representative of the chemistries that would be submitted in the future. From 1998 through 2017, there were a total of 181 conventional pesticides registered by the USEPA. The USEPA’s ChemSearch database (https://iaspub.epa.gov/apex/pesticides/f?p=chemicalsearch:1) was searched for publically-available documents for these 181 chemicals that contain information for the avian acute oral and sub-acute dietary tests; including: ecological risk assessments (ERA), problem formulations (PF), preliminary risk assessments (PRA), and final work plans (FWP).

### 2.2. Dataset collection and characterization

Each document was reviewed for relevant data, and the data were collected by a systematic method following a detailed reporting framework which outlined the information to be recorded for each chemical. The following information was collected for 119 chemicals, and then reviewed for accuracy and completeness: chemical target, chemical class, mode of action, empirical formula, CAS reference number, Simplified Molecular Input Line Entry System (SMILES) code, molecular weight, Log K_ow_, toxicity values (LD_50_ or LC_50_), RQ values, and any additional study observations that were recorded for each avian test. The chemical class and mode of action information was collected by first separating the chemicals by target, and then searched for standard identification by the following Resistance Action Committee: Herbicide (HRAC) (HRAC, 2018), Fungicide (FRAC) (FRAC, 2018), and Insecticide (IRAC) (IRAC, 2018). If a chemical had available endpoints from more than one acute oral study and more than one sub-acute dietary study, only the acute oral study and the sub-acute dietary study with the most sensitive endpoint was included in the analysis.

All available documents were exported and reviewed for relevant toxicity and physicochemical information. The ERA and PRA documents typically contained toxicity information for both acute and sub-acute endpoint values, as well as calculated RQ values. If the PF and FWP documents contained definitive LD_50_ and LC_50_ values, but not RQ values, then toxicity information from data evaluation records (DERs), reviews conducted by the USEPA of studies submitted by registrants, were examined to ensure all relevant information was collected for analysis. DERs were consulted for two of the 119 chemicals; including cyazofamid and fenpropimorph. Finally, if the documents reported chemical toxicity by limit test (i.e., LD_50_ ≥ 2000 mg a.i./kg-bw for acute oral, or LC_50_ ≥ 5000 mg a.i./kg-diet for sub-acute dietary), and no additional studies were requested for the chemical, then no avian risk is presumed and these data were included in the analysis.

### 2.3. Approach to analysis

The chemicals were further assessed by comparing acute oral versus sub-acute dietary toxicity categories. Chemicals were separated by the number of those with a limit test or a definitive test (a study comprised of a minimum of five levels of a test substance plus control that is used to establish a dose response), for the analysis of toxicity data used to calculate RQ values. Chemicals reported to have no toxicity via limit tests are classified as practically non-toxic and are generally not considered to be of concern to avian populations. Risk estimation was conducted by comparing the calculated RQ values to the LOC for each test. The toxicity data used to calculate RQs are generally from the most sensitive species tested in each taxon. For example, for a specific pesticide, if the RQs based on the LD50 from a mallard duck study are higher than the RQs based on the LD_50_ from a bobwhite quail study, the RQs based on the mallard duck endpoint would be used to calculate the RQ value that would be compared to the acute LOC. The risk presumptions for terrestrial animals are reported by the USEPA for RQ values greater than or equal to the LOC (EPA, 2017b). Generally, the highest RQ for each taxon drives the risk conclusion for that taxon.

Acute and sub-acute RQ values for birds are typically calculated by the USEPA OPP using the Terrestrial Residue EXposure (T-REX) model (EPA, 2017c). T-REX calculates the residues on avian (and mammalian) food items along with the dissipation rate of a chemical applied to foliar surfaces for single or multiple pesticide applications. The T-REX model adjusts toxicity values based on relative body weight of animals being assessed compared to the animal used in toxicity studies, and it is used to calculate acute oral and dietary RQ values for granular applications and seed treatments based on residue and dissipation rate calculations (https://www.epa.gov/pesticide-science-and-assessing-pesticide-risks/models-pesticide-risk-assessment#terrestrial). The results of the T-REX calculations are presented by various weight classes and dietary categories (for foliar uses) for generic birds and mammals. The weight classes used for calculating EEC equivalent doses based on estimated dietary concentrations include: small (20 g), medium (100 g), and large (1000 g), and T-REX adjusts acute values based on the relative body weight of the animal being assessed compared with the animal used in the toxicity studies.

The highest RQ value from each weight class was used to compare the avian sub-acute dietary versus the acute oral exposure. Definitive toxicity tests were used to calculate RQ values, and limit tests were presumed to not be an avian risk concern for chemicals that were classified as practically non-toxic (i.e., LD50 ≥ 2000 mg a.i./kg-bw for acute oral, or LC50 ≥ 5000 mg a.i./kg-diet for sub-acute dietary). Additionally, the chemical class and mode of action data were carefully reviewed for each chemical to examine potential trends of toxicity for the avian acute oral and sub-acute dietary tests. Data were analyzed and graphed using R studio v3.2.2, and Microsoft Office Professional Plus 2016.

## 3. Results

Of the 181 pesticides searched on the USEPA OPP Pesticide Chemical Search website, 119 chemicals had publicly available risk assessment documents of which 41 RQ values were used for the acute oral and sub-acute dietary RQ comparative analyses described below.

### 3.1. Limit and definitive tests

For 87 chemicals, the endpoints are from limit tests for both the acute oral and sub-acute dietary studies. It is important to note that some chemicals tested by the definitive tests did not have reported RQ values available due to the document source. To ensure that the definitive tests were not considered risk drivers, definitive toxicity data was checked for reporting accuracy (i.e., check an acute oral test with an LD_50_ reported as 2000 mg a.i./kg-bw instead of > 2000 mg a.i./kg-bw). There were a total of 6 pesticides missing toxicity information for which the USEPA reviewed the following documents in order to determine avian study outcome: data evaluation records (DERs), previous risk assessments, new use assessments, and problem formulations. These chemicals were found to either have a new use registration that did not require the acute avian test submission, or were found to have been conducted as a limit test, and therefore no risk is presumed.

### 3.2. Mode of action

Table 1 shows chemical assignment to respective chemical classes by target for the 181 chemicals. Chemicals used for the RQ value comparison are italicized. Of the 181 chemicals searched in the OPP Pesticide Chemical Search website, 62 were not included in the above analyses because public documents containing information on acute oral and sub-acute dietary studies were not available through the USEPA’s ChemSearch database. A review of the chemical modes of action (MOA) was completed to determine whether the MOAs for the 62 chemicals was covered by one of the 119 pesticides for which the comparison of RQs was completed (i.e., did the chemical share a chemical class with one of the chemicals included in the analyses?). Only 7 of the 62 chemicals did not share a chemical class with a pesticide included in the RQ comparison, and 6 of these 7 chemicals had unique MOAs (Supplemental Table 1). Therefore, in the majority of unevaluated cases (87%), the chemical class was represented by an analogue in the RQ analysis.

### 3.3. RQ value comparison

**Fig. 1.**
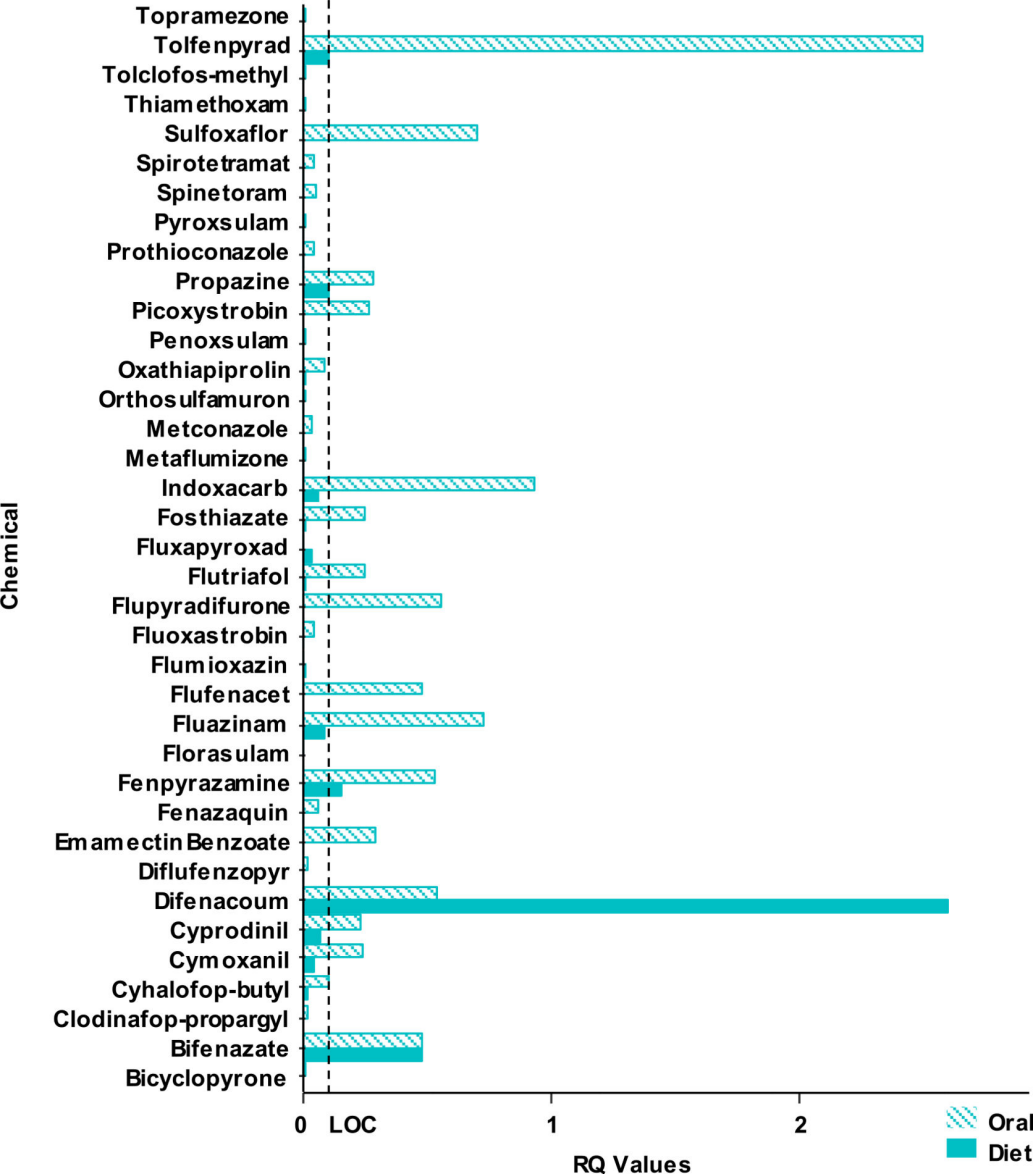
Bar graph used to illustrate acute oral versus sub-acute dietary RQ values. RQ values greater than 3 are removed as outliers for figure resolution: Acetamiprid (oral = 3.83, diet = 0.03), Benzovindiflupyr (oral = 4.6, diet = 0.01), Fluensulfone (oral = 7.25, diet = 0.15), Furfural (oral = 309.97, diet = na). Avian LOC: ≥ 0.10 for listed species, ≥ 0.50 acute risk, and ≥ 1.0 chronic risk.

**Fig. 2.**
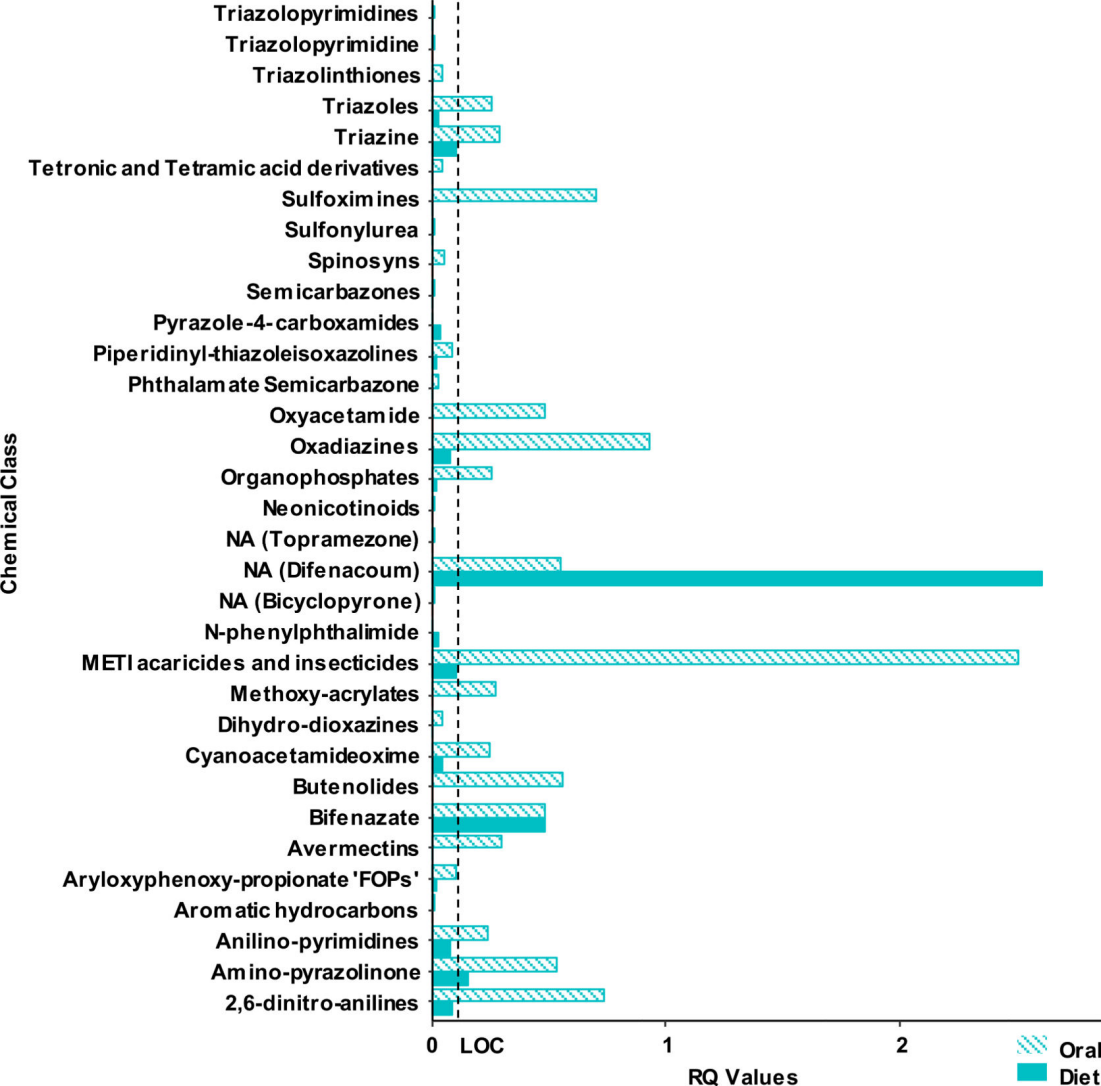
Comparison of the highest RQ values recorded for acute oral versus sub-acute dietary test by chemical class. Chemical classes containing RQ values greater than 3 are removed as outliers for figure resolution: Acetamiprid (oral = 3.83, diet = 0.03), pyrazole-4-carboxamides (oral = 4.6, diet = 0.01), Fluensulfone (chemical class = NA, oral = 7.25, diet = 0.15), Furfural (chemical class = NA, oral = 309.97, diet = NA). Avian LOC: ≥ 0.10 for listed species, ≥ 0.50 acute risk, and ≥ 1.0 chronic risk. NA = not available.

RQ values were used to compare the avian acute oral and sub-acute dietary endpoints because they take into account exposure estimates. Overall, 41 chemicals contained RQ value information and were used for the oral versus dietary test comparison (Supplemental Table 2). These RQ values were used for the study comparison in Fig. 1 which illustrates a bar graph of the highest reported RQ value, which was consistently found to be in the small weight class (20 g), for the acute oral and the sub-acute dietary tests. Only one chemical, difenacoum, had a sub-acute dietary RQ value greater than the acute oral RQ value. However, both the dietary and oral tests for difenacoum reported RQ values greater than the LOC, thus indicating a risk concern for birds. Therefore, the risk outcome for difenacoum would have been the same regardless of which endpoint was used to calculate risk. Fig. 2 represents a bar graph of the highest reported RQ value for the acute oral and the sub-acute dietary tests by chemical class. Note, both Figs. 1 and 2 do not include RQ values greater than 3 in order to show higher resolution of differences between the two endpoints. In general, sub-acute dietary RQ values are shown to be less than the LOC of 0.10 across most chemical classes, unlike the acute oral RQ values.

## 4. Discussion

The results of the current analysis indicate that in more than 99% of the pesticides included in the analysis, the highest RQ values for exposure in birds were based on the acute oral endpoint, not the sub-acute dietary test. Of the 119 chemicals evaluated for avian acute oral and sub-acute dietary tests, only one chemical, the anticoagulant rodenticide difenacoum, was found to have a sub-acute dietary RQ value greater than the acute oral RQs. The high RQ value of anticoagulants from avian sub-acute dietary exposure is due to increased likelihood of bioaccumulation from repeat dosing of chemicals with a high Log K_OW_. The Log K_OW_ provides physiochemical information for how a chemical will partition between octanol and water, where a Log K_OW_ >4 is considered hydrophobic, and thus will have a greater affinity to fats and lipids (EPA, 2012c). Additional physiochemical and metabolic information should be considered during the assessment of pesticide toxicity, including the following conditions in which the sub-acute dietary test would be used for risk assessment: compounds with a MOA that suggests delayed toxicity through accumulation of damage; compounds with high potential to bioaccumulate (high lipophilicity, low metabolism rate, low excretion rates); compounds with very high molecular weights or other absorption data that suggest gastric uptake by mechanisms other than simple diffusion; compounds that result in regurgitation in acute oral tests. The anticoagulant difenacoum contains a Log K_OW_ of 6.09 (pH 6.5, 20 °C) and a molecular weight of 444.5 g/mol, that may in part explain its greater toxicity via repeat sub-acute dietary exposure compared to single dose acute oral exposure.

Pesticide regulation and requirements to conduct the avian sub-acute dietary test vary by country. As of 2013, the European Union (EU) does not routinely require the avian sub-acute dietary test (Commission, 2013). According to the EU regulation, the sub-acute dietary test is only to be conducted to determine intrinsic toxicity through dietary exposure when either there is suggested toxicity by the MOA, or if the mammalian results generate a lower toxicity value through dietary testing than by oral testing. However, the United States and Canada still require both the oral and dietary toxicity testing on non-target terrestrial species, including bobwhite quail and mallard duck (Canada, 2004). If the USEPA granted waivers for the sub-acute dietary test, hundreds to thousands of birds and approximately $83,000 USD (~ $8300 per sub-acute dietary test) would be saved every year in this one test alone. Ultimately, the results from this retrospective review can be used for further consideration of routine requirements for avian testing.

## 5. Conclusion

The retrospective review comparing avian acute oral versus sub-acute dietary RQ values of 119 pesticides registered with the USEPA from 1998 to 2017 has demonstrated that there are no cases in which the sub-acute test identifies risk that is not also identified in the acute oral test. While the sub-acute RQ value was greater than the acute oral for the anticoagulant rodenticide difenacoum, both the acute oral and the sub-acute dietary RQ values were greater than the endangered species LOC of 0.10. Thus, the sub-acute dietary test was not a risk driver, suggesting that this test could be waived for all pesticides that do not fall into the exemption criteria described above (e.g., compounds with delayed toxicity, a high potential to bioaccumulate, a high molecular weights and/or those that result in regurgitation). Ultimately, the sub-acute dietary test waiver can spare hundreds to thousands of birds per year, and support the USEPA’s goal of reducing tests on animals (EPA, 2018b).

## Supplementary Material

Chemical List

MOA

## Figures and Tables

**Table 1.** 181 chemicals listed by chemical class (**bold**) for A) insecticide (http://www.irac-online.org/documents/moa-dassification/), B) herbicide (http://hracglobal.com/tools/dassification-lookup), and C) fungicide (http://www.frac.info/docs/default-source/publications/frac-code-list/frac_code_list_2018-final.pdf?sfvrsn=6144b9a_2), and D) other. Chemicals used for RQ comparison are *italicized*.

A	INSECTICIDE		B	HERBICIDE		C	FUNGICIDE			D	OTHER
	**Acequinocyl**	**Neonicotinoids**		**Alkylazines**	**Pyrimidinyl(thio) benzoate**		**2,6-dinitro-anilines**	**Methoxy-carbamates**	**Toluamides**		Prohexadione Calcium
	Acequinocyl	*Thiamethoxam*		Indaziflam			*Fluazinam*	Pyraclostrobin	Zoxamide		Oxalic acid
		Clothianidin			Bispyribac Sodium						Nicarbazin
	**Acetamiprid**	Dinotefuran		**Aryloxyphenoxy-propionate ‘FOPs’**			**Acylalanines**	**Morpholines**	**Triazoles**		Iodomethane
	*Acetamiprid*	Thiacloprid			**Sulfonylamino carbonyl-triazolinone**		Benalaxyl-M	Fenpropimorph	*Flutriafol*		n-methylneodecanamide
				*Clodinafop-propargyl*					Ipconazole		Mammalian Gonadotropin Releasing Hormone
	**Avermectins**	**Oxadiazines**		*Cyhalofop-butyl*	Flucarbazone-sodium		**Amino-pyrazolinone**	**N-methoxy-pyrazole-carboxamides**	*Metconazole*		Ammonium Nitrate
	*Emamectin Benzoate*	*Indoxacarb*			Thiencarbazone-methyl		*Fenpyrazamine*		Bromuconazole		Calcium Nitrate
	Milbemectin			**Arylpicolinate**	Propoxycarbazone-Sodium			Pydiflumetofen	Epoxiconazole		Cuprous Chloride
		**Phenylpyrazoles**		Halauxifen-methyl			**Anilino-pyrimidines**		Tetraconazole		EH-2001
	**Benzoylureas**	Ethiprole			**Sulfonylurea**		*Cyprodinil*	**N-phenylcarbamates**	Triticonazole		Oxysilver Nitrate
	Novaluron			**Cyclohexanedione ‘DIMS’**	Flazasulfuron		Mepanipyrim	Diethofencarb			Potassium tri-iodide
	Noviflumuron	**Phosphides**			Foramsulfuron		Pyrimethanil		**Triazolinthiones**		S-Dimethenamid
	Flufenoxuron	Phosphine Gas		Tralkoxydim	Imazosulfuron			**Oxazolidine-diones**	*Prothioconazole*		Sodium nitrite
	Lufenuron				Mesosulfuron-methy1		**Aromatic hydrocarbons**	Famoxadone			Tepraloxydim
	Teflubenzuron	**Pyrethroids/Pyrethrins**		**Isoxazole**	*Orthosulfamuron*		*Tolclofos-methyl*		**Triazolo-pyrimidylamine**		Zona-Stat
		Etofenprox		Isoxaflutole	Sulfosulfuron			**Oximino-acetates**			*Fluensulfone*
	**Beta-ketonitrilederivatives**	Imiprothrin			Trifloxysulfuron-sodium		**Aryloxyquinoline**	Kresoxim-methyl	Ametoctradin		*Fosthiazate*
		Flumethrin		**Long Chain Fatty Acid Inhibitor**	Ethametsulfuron Methyl		Quinoxyfen	Trifloxystrobin			*Furfural*
	Cyflumetofen								**Valinamide carbamates**		Demiditraz
		**Pyridalyl**		Pyroxasulfone	**Thiadiazole**		**Benzophenone**	**Phenylacetamide**	Benthiavalicarb-isopropyl		Forchlorfenuron
	**Bifenazate**	Pyridalyl			Fluthiacet-methyl		Metrafenone	Cyflufenamid	Iprovalicarb		PT807 (Ecolyst)-HCl
	*Bifenazate*			**N-phenylphthalimide**							VCD and Triptolide
		**Pyridine azomethine**		*Flumioxazin*	**Triazine**		**Benzothiadiazole**	**Phenyl-oxo-ethylthiophene amide**	**NA**		*Difenacoum*
	**Buprofezin**	Pymetrozine			*Propazine*		Acibenzolar-s-methyl		Macleaya extract chloride		alpha-Chlorohydrin
	Buprofezin	Pyrifluquinazon		**Other (PPO)**				Isofetamid			Acetaminophen
				Flufenpyr-ethyl	**Triazolinone**		**Benzoylpyridine**				Dimethyl disulfide
	**Butenolides**	**Semicarbazones**			Amicarbazone		Pyriofenone	**Picolinamides**			
	*Flupyradifurone*	*Metaflumizone*		**Oxyacetamide**	Carfentrazone-ethyl			Amisulbrom			
				*Flufenacet*	Azafenidin		**Cinnamic acid amides**				
	**Cypermethrin**	**Spinosyns**					Dimethomorph	**Piperidines**			
	alpha-Cypermethrin	*Spinetoram*		**Phenylpyrazole**	**Triazolopyrimidine**			Fenpropidin			
				Pyraflufen-ethyl	Cloransulam-methyl		**Cyanoacetamideoxime**	Spiroxamine			
	**Diacylhydrazines**	**Sulfoximines**			*Florasulam*		*Cymoxanil*				
	Methoxyfenozide	*Sulfoxaflor*		**Phenylpyrazoline**	*Penoxsulam*			**Piperidinyl-thiazoleisoxazolines**			
				Pinoxaden	*Pyroxsulam*		**Cyano-imidazole**				
	**Diamides**	**Tetronic and Tetramic**			Diclosulam		Cyazofamid	*Oxathiapiprolin*			
	Chlorantraniliprole	*Spirotetramat*		**Phthalamate Semicarbazone**							
	Cyantraniliprole	Spirodiclofen			**Triketone**		**Dihydro-dioxazines**	**Pyrazole-4-carboxamides**			
	Flubendiamide	Spiromesifen		*Diflufenzopyr*	Mesotrione		*Fluoxastrobin*				
								*Benzovindiflupyr*			
	**Etoxazole**	**NA**		**Pyridine carboxylic acid**	**NA**		**Dinitrophenyl-crotonates**	*Fluxapyroxad*			
	Etoxazole	Metofluthrin			Aminocyclopyrachlor		Meptyldinocap	Penflufen			
		Momfluorothrin		Aminopyralid	*Bicyclopyrone*			Penthiopyrad			
	**Flonicamid**	Lithium (perfluorooctane)-Sulfonate		Fluroxypyr	Iodosulfuronmethyl Sodium		**Ethylamino-thiazolecarboxamide**	Sedaxane			
	Flonicamid				Pyrasulfatole			Iospyrazam			
				**Pyrimidinediones**	Tembotrione		Ethaboxam				
	**METI acaricides and insecticides**			Saflufenacil	*Topramezone*			**Pyridine carboxamides**			
				Butafenacil			**Hexopyranosylantibiotic**	Nicobifen			
	*Fenazaquin*						Kasugamycin				
	*Tolfenpyrad*							**Pyridinyl-ethylbenzamides**			
	Fenpyroximate						**Hydroxyanilides**				
	Tebufenpyrad						Fenhexamid	Fluopyram			
							**Imidazolinones**	**Pyridinylmethyl benzamides**			
							Fenamidone				
								Fluopicolide			
							**Mandelic acid amides**				
							Mandipropamid	**Quinazolinone**			
								Proquinazid			
							**Methoxy-ecetamide**				
							Mandestrobin	**Quinones**			
								Dithianon			
							**Methoxy-acrylates**				
							*Picoxystrobin*	**Sulfamides**			
								Tolylfluanid			
